# The Relationship Between Clinical Features of Ischemic Stroke and miRNA Expression in Stroke Patients: A Systematic Review

**DOI:** 10.3390/neurolint17040055

**Published:** 2025-04-09

**Authors:** Katarzyna Prus, Konrad Rejdak, Federico Bilotta

**Affiliations:** 1 Department of Neurology, Stroke, and Early Post-Stroke Rehabilitation, University Clinical Hospital No. 4, 20-954 Lublin, Poland; k.rejdak@umlub.pl; 2 Department of Neurology, Medical University of Lublin, 20-954 Lublin, Poland; 3 Department of Anesthesiology, Intensive Care and Pain Management, “Sapienza” University of Rome, 00184 Rome, Italy; federico.bilotta@uniroma1.it

**Keywords:** ischemic stroke, miRNA, stroke severity, neurological deficit, lesion volume, inflammation

## Abstract

**Background/Objectives:** Ischemic stroke remains a leading cause of death and disability worldwide. Despite significant progress in reperfusion therapy, the optimal ischemic stroke management strategy has not been developed. Recent studies demonstrate that microRNA may play an essential role in the pathophysiology of ischemic stroke and its possible potential to be a treatment target point. The proposed systematic review aimed to report the relationship between IS’s clinical severity and miRNA expression. Secondary outcomes included infarct volume, systemic inflammatory markers, and prognosis, as well as additional features such as stroke subtype, comorbidity, and risk of subsequent stroke in correlation to miRNA expression. **Methods**: We have performed a systematic search of database resources according to PRISMA statement guidelines. Twenty-seven studies on a total number of 3906 patients were assessed as suitable for the present SR. Included studies analyzed the expression of 30 different miRNA fragments. **Results**: After investigating available data, we have identified a set of possible miRNA fragment candidates that may be used in stroke diagnostics and have the potential to be a base for the development of future treatment protocols. **Conclusions**: Studies included in the presented SR indicate that miRNA expression may be significantly associated with clinical severity, infarct volume, and inflammation in ischemic stroke. More prospective, properly designed protocols with consistent methods of miRNA testing and optimized clinical assessment are needed to confirm the role of miRNA expression in the course of a stroke.

## 1. Introduction

Ischemic stroke (IS) remains a leading cause of death and disability worldwide [1]. Despite decades of research into risk factors, therapies, and preventative measures, the optimal management strategy for the treatment of IS has not been developed. As demonstrated in recent studies, microRNA (miRNA) may play a vital role in the pathophysiology of IS [2]. Alterations in miRNA have been proven to impact detrimental processes at different stages of ischemic brain injury, such as neuroinflammation, oxidative stress reactions, blood–brain barrier permeability [3,4,5], and neuroplasticity in post-stroke recovery [6,7]. Numerous miRNA-based therapies are undergoing clinical trials for different pathologic conditions, indicating their possible therapeutic significance [8]. At the same time, growing evidence confirms that miRNAs can serve as biomarkers that help predict, diagnose, and evaluate the prognosis in IS patients [4,9] and become a potential therapeutic target in ischemic brain injury [10].

Despite recent advances in reperfusion treatment, IS’s clinical and anatomical severity still strongly correlates with patients’ future outcomes [11]. Standardized and valid laboratory tests evaluating the extent of ischemic brain injury are missing. The assessment of miRNA expression could potentially serve as a diagnostic tool in monitoring IS progression, predicting patient recovery, and, finally, enabling the search for novel treatment strategies.

This systematic review aims to present clinical evidence for the relationship between miRNA expression and anatomical and clinical features in IS.

## 2. Materials and Methods

A systematic search of database resources, PubMed, Science Direct, and Cochrane Library, was independently performed by two researchers according to the Preferred Reporting Items for Systematic Reviews and Meta-analyses (PRISMA) statement guidelines. The study has been registered in the International Prospective Register of Systematic Reviews (PROSPERO) database (registration number CRD42023472594) [12].

**Figure 1:**
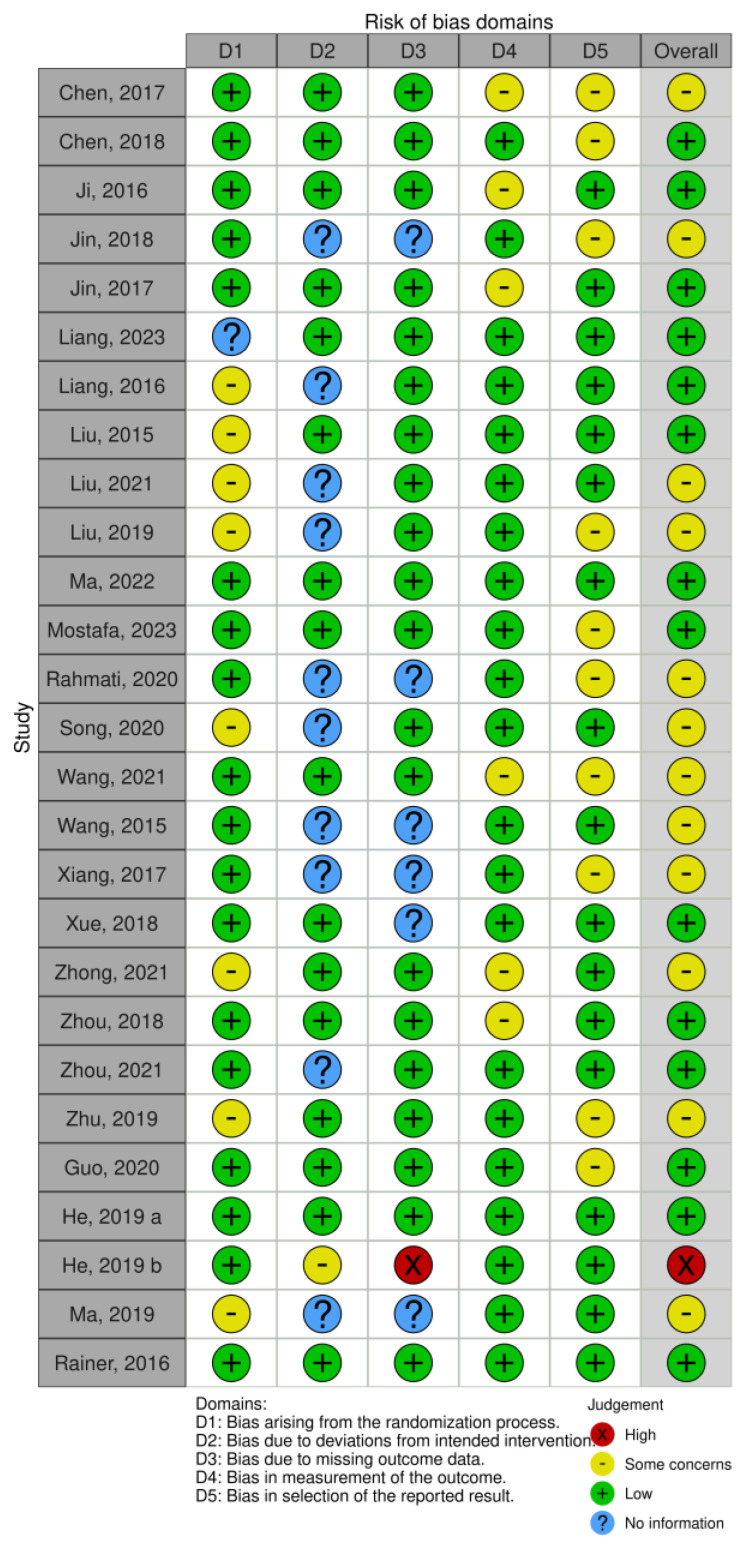
Risk of bias assessment for the included studies [14].

The following keywords were used: microRNA AND ischemic stroke OR ischemic brain injury. The applied filters were studies published until April 2024, full-length articles (no abstracts), human adult population (older than 18 years), and English language. The literature search included references from reviews and original articles. Duplicates were eliminated [13]. Case-control, cross-sectional, cohort studies, and case series that included more than five patients and reported the correlation between plasma and serum miRNA expression in ischemic stroke patients and IS clinical and anatomical characteristics were eligible for the present SR. Data extraction also included other reported features associated with miRNA in IS patients, such as level of inflammatory parameters, prognosis, stroke etiology type, and IS recurrence rate. Case reports, systematic reviews, and meta-analyses were excluded from the search. The risk of bias assessment was based on the RoB2 assessment tool. Bias items were assessed separately for each included study (Figure 1).

The primary outcome was to report on the relationship between IS clinical severity and miRNA expression in stroke patients. The secondary outcomes included infarct volume, systemic inflammatory markers, prognosis, and additional features such as stroke subtype, comorbidity, and the risk of subsequent IS in correlation to miRNA expression in stroke patients.

## 3. Results

**Figure 2:**
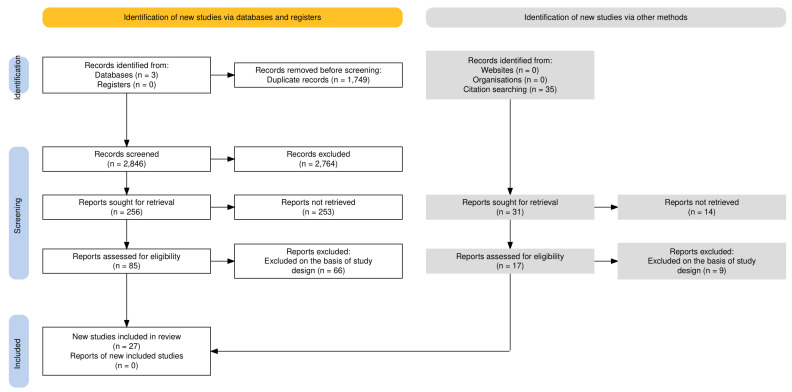
PRISMA flow chart [15].

The database search led to 4595 matching articles. After screening for eligibility, 4568 articles were excluded, as they did not match the inclusion criteria (Figure 2). Twenty-seven studies, including a total number of 3906 patients, were assessed as suitable for the present SR (four prospective cohort studies and 23 prospective case-control studies) (Table 1). In the selected studies, expression of plasma and serum miRNA in IS patients was reported in correlation with six outcomes: clinical severity; infarct volume; systemic inflammatory markers; prognosis; stroke etiology subtype; and risk of stroke recurrence. The included articles assessed the expression of 30 different miRNA fragments.

### 3.1. The Relationship Between Clinical Severity of IS and miRNA Expression

All the included studies (27 articles, 3906 patients) report on the relationship between clinical severity of IS and expression of selected miRNA [16,17,18,19,20,21,22,23,24,25,26,27,28,29,30,31,32,33,34,35,36,37,38,39,40,41,42]. Clinical severity was measured using the National Institutes of Health Stroke Scale (NIHSS) at admission as well as the modified Rankin Scale (mRS) and Glasgow Outcome Scale (GOS). Studies analyzing the relationship between the clinical severity of IS and miRNA expression included the assessment of 30 different miRNA fragments (Table 2). The majority of the selected studies (24/27 studies, 3430 patients) used NIHSS to measure clinical severity [16,17,18,19,20,21,22,23,24,25,26,27,28,29,31,32,33,34,35,37,38,39,40,41]. In eight studies including 804 patients, clinical assessment was performed using mRS [25,27,28,30,31,39,40,42]. In one study on 216 patients, GOS assessment was used to measure clinical severity [36].

A positive correlation between clinical severity of IS and increased miRNA expression was reported in 14 studies that evaluated 15 different miRNA fragments: miR-9, miR-101, miR-124, miR-125b-5p, miR-128, miR-134, miR-143, miR-146b, miR-185, miR-206, miR-218, miR-222, miR-223, miR-488, and miR-602 (Table 2) [16,17,18,19,20,21,25,27,28,33,35,37,39,40]. The most pronounced positive correlation was reported for miR-125b-5p (three studies: one case-control and two cohort studies, including 218 stroke patients), miR-218 (two studies: both case-control, including 254 stroke patients), and miR-143 (one case-control study with 170 stroke patients).

A negative correlation between clinical severity and the level of miRNA expression was reported in 12 studies including 12 different miRNA fragments: miR-9, miR-16, miR-21, miR-24, miR-29b, miR-34a-5p, miR-124, miR-126, miR-130, miR-152-3p, miR-378, and miR-497 [19,20,22,23,24,26,29,31,34,38,43]. The negative correlation was most identifiable for miR-29b (three case-control studies, including a sum of 288 patients), miR-126 and miR-130 (two case-control studies on 254 patients), and miR-21 (one case-control study with 170 patients)

Extracted data on the relationship between the clinical severity of IS and miRNA expression were mixed in the cases of miR-9, miR124, and miR-128, indicating positive, negative, or no correlation, depending on the study.

### 3.2. The Relationship Between Infarct Volume at Admission and miRNA Expression

The correlation between infarct volume and miRNA expression was assessed in 12 studies, including 1390 patients [16,17,18,22,23,25,27,31,35,36,40,41]. Infarct volume was measured using magnetic resonance imaging (MRI) performed at admission within 24 to 72 h of IS onset, depending on the protocol. Studies that included infarct volume as an outcome measure assessed the expression of 11 different miRNA fragments. (Table 2)

A positive correlation between the IS infarct volume and miRNA expression was reported for miR-9, miR-124, miR-125b-5p, miR-128, miR-134, miR-146b, and miR-206 [17,18,27,35,40]. The association was most pronounced for miR125b-5p (two studies: one case-control, one cohort, 134 patients).

Three studies assessing the relationship between the infarct volume and the expression of miR-9, miR-29b, miR-34a-5p, and miR-124 reported a negative correlation [23,24,32].

Four studies on miR-93, miR-124, miR-128, and miR-223 reported no significant association between IS infarct volume and miRNA expression level [16,25,36,41].

Data on the association between infarct volume and miR-9, miR-124, and miR-128 expression were inconsistent.

### 3.3. The Relationship Between Systemic Inflammatory Markers and miRNA Expression

A total of 11 studies included in this SR analyzing the data from 1906 subjects reported on the relationship between the level of systemic inflammatory markers or oxidative stress indicators and the expression of miRNA fragments. These studies reported on the expression of 10 different miRNA fragments (Table 2). Analyzed laboratory parameters included CRP, IL-1 beta, IL-6, IL-2, IL-8, IL- 10, IL-17, IL-22, TNF-α, SOD, and MDA concentration [17,18,19,23,24,33,34,35,36,37,41] measured throughout hospitalization; timing differed depending on the protocol.

Six of the included studies on miR-9, miR124, miR-134, miR-143, miR-146, and miR-497 reported a positive correlation between elevated levels of inflammatory and oxidative stress parameters and miRNA expression [17,18,33,34,35,37].

In five studies on miR-9, miR-21, miR-93, miR-124, miR-126, and miR-130, the negative correlation between the level of systemic inflammatory markers and miRNA expression was confirmed [19,23,24,36,41].

Data regarding the relationship between the level of systemic inflammatory markers and the expression of miR-9 and miR-124 were inconsistent.

### 3.4. The Relationship Between Prognosis and miRNA Expression

Three studies assessing four different miRNA fragments (Table 2) reported a correlation between patients’ prognosis after IS and miRNA expression [35,36,38]. A worse prognosis was defined as mRS > 2 at 3 months of follow-up [35,38] or elevated GOS scores at discharge [36].

One case-control prospective study confirmed the association between elevated expression of miR-134 and worse prognosis [35].

Two studies reported the relationship between poor prognosis and low miR-9, miR-24, and miR-124 expression in IS patients [36,38].

### 3.5. The Relationship Between Stroke Etiology Subtype and miRNA Expression

Four included studies on four different miRNA fragments (Table 2) analyzed the correlation between specific etiology subtypes of stroke and the level of miRNA expression [16,21,30,36].

Three studies reported no significant correlation between stroke subtype and miRNA expression (miR-124, miR-223, miR-488).

One of the included studies demonstrated a significant decrease in miR-152-3p during large-artery atherosclerosis.

### 3.6. The Relationship Between the Risk of Stroke Recurrence and miRNA Expression

Two case-control prospective studies, including three different miRNA fragments, assessed the relationship between the risk of subsequent stroke episodes and the expression of selected miRNA [19,24]. Presented data reported a negative correlation between the risk of stroke recurrence and the level of miRNA expression (miR-21, miR-126, and miR-130).

## 4. Discussion

The present SR reports available data on the relationship between miRNA expression in stroke patients and selected IS features, such as clinical severity, infarct volume, systemic inflammatory and oxidative stress markers level, prognosis, IS etiology subtype, and the risk of stroke recurrence. This work aimed to analyze the role of miRNA in IS and to identify miRNA fragment candidates for diagnostic biomarkers and possible future treatment targets. The results of the included studies reported on both enhanced and downregulated miRNA expression in IS patients; therefore, the correlation between miRNA expression and clinical severity, infarct volume, systemic inflammatory markers, prognosis, and the risk of stroke recurrence varied for different miRNA fragments (Table 2).

The binary correlations between miRNA expression and selected clinical outcomes in IS patients suggest that different miRNA fragments may be involved in various pathological and protective reactions following ischemic brain injury. Numerous researchers investigated the role of specific miRNA fragments in stroke. Research on animal studies reports that miRNA expression in the course of ischemic injury may be related to energy failure, excitotoxicity, inflammation, oxidative stress, cell death, and brain–blood barrier disruption [2]. As a result, these may affect both the lesion volume and neurological deficit [43]. Accumulative data indicate that particular miRNAs play a significant part in the development of inflammatory responses in the course of stroke [3]. The expression of miR-181c, miR-216a, miR-3437b, and miR-126-3p or -5p are reported to be linked with the post-ischemic CNS levels of TNF [3,44,45]. A part of the inflammatory response to ischemia is the upregulation of the expression of adhesion molecules which facilitates adherence of leukocytes and increases BBB permeability [46]. Various miRNAs are engaged in stroke-induced BBB disruptions. Overexpression of miR-150 by regulating claudin-5 expression and endothelial cell survival can decrease BBB hyperpermeability and, as a consequence, infarct volume and neurological deficit [47]. There is also data on BBB-protective effects of miR-130a and miR-155, due to increasing the expressions of occludin and ZO-1 [48,49]. miRNA-mediated pathologies such as energy failure, excitotoxicity, oxidative stress, and inflammation can induce regulated cell death in the penumbra area and, through this mechanism, extend the neurovascular damage [50] Following cerebral ischemic injury, particular miRNAs (miR-214, miR-128) were proven to regulate pro-apoptotic genes by decreasing pro-apoptotic protein Bax and increasing the anti-apoptotic protein Bcl-2’s expression [2]. Most available human studies report solely on the expression levels of particular miRNAs in IS patients in comparison to healthy subjects [51]. A comprehensive review of the correlation between miRNA and clinical or anatomical features of stroke is scarce.

A recent SR and meta-analysis from Burlacu et al. reported the relationship between miRNA expression and post-stroke recovery [7]. In this article, the authors summarized available data on miRNA expression variability, which is related mainly to long-term neurological improvement. As a result of a comprehensive analysis, the authors suggested that miR-9 and miR-29b (isolated from neutrophils), as well as serum miR-124 and miR-125b, are the most promising biomarkers for follow-up studies. The results are only partially consistent with the present SR, probably due to different approaches to the timing of miRNA testing and clinical assessments. Similarly to Burlacu’s work, studies included in the present SR supported the data on miR-9, miR-29b, miR-124, and miR-125b relevance in IS. However, our analysis revealed contradictory findings on the correlation between miR-9 and miR-124 expression and the clinical severity of IS. That may indicate that these fragments are not the best candidates to include in diagnostic panels for miRNA testing in IS patients. Burlacu’s SR was aimed to assess the correlation between miRNA expression and the neurologic deficit and focused on miRNAs as prognostic biomarkers. In our SR we have included studies that analyzed more clinical features associated with stroke severity (lesion volume, inflammatory markers). It is worth mentioning that in many cases all these outcomes appeared to be correlated with particular miRNA expression levels. This type of approach addresses a wider scope of pathologies in an acute period of ischemic stroke and at the same time reflects a possible role of miRNAs as mediators of pathological processes (apoptosis, inflammation, blood–brain barrier permeability). In this review, we intend to indicate the direction for designing future research protocols and identify clinical correlations that can give ground for utilizing miRNA both as possible diagnostic biomarkers and treatment targets. The SR by Burlacu et al. identified multiple shortcomings that should be addressed before including miRNA-based diagnostics in clinical practice and preparing protocols for further research. The main reported issues were the lack of studies investigating long-term outcomes, the possible discrepancy between miRNA expression in the plasma and serum, the high risk of bias, and the low sample size of many studies.

Studies included in the present SR analyzed multiple numbers of miRNA fragments in relation to important features of IS, such as clinical severity, infarct volume, inflammation, prognosis, etiology, and the risk of stroke recurrence. The protocols of these assessments were substantially alike. In the predominance of studies, blood samples for miRNA expression testing were collected just after hospital admission or within 24 h from IS onset. The clinical assessment of neurological deficit and brain imagining were conducted during the same period. Therefore, most of the clinical outcomes and infarct volumes reported in the included studies represent the acute phase of stroke, in which the central part of IS-induced brain injury takes place. It is worth noting that miRNA expression during this critical phase is probably associated with acute consequences of ischemia, such as inflammation, excitotoxicity, oxidative stress, and apoptosis [43]. Possible candidates for biomarkers assessed during this “time window” indicate the extent and the severity of brain damage. This may lead the way for future search for miRNA-based therapies targeting the acute phase and preventing the progression of ischemic injury. The timing of sample collection for miRNA expression assessment may be strongly associated with the role of detected fragments in stroke pathology. Edwardson et al. addressed this problem in an interesting SR article on plasma microRNA expression in correlation to markers of post-stroke recovery [52]. Researchers suggest plasma miRNAs may become promising biomarkers of spontaneous biological recovery following stroke. However, the period in which blood samples for miRNA testing were obtained is highly significant. Authors stress the importance of longitudinal assessments, stating that whereas fragments detected during the hyperacute phase are most probably the indicators of brain tissue damage, the miRNA fragments involved in the chronic phase may be associated with post-stroke recovery, including neuroplasticity and neuroregeneration. This shows that despite promising data on the role of miRNA expression in the course of IS, its significance is most probably strictly related to the timing of sample collection and the stage of the disease

Part of the studies included in the present SR assessed the neurologic deficit in long-term follow-up [25,28,31,34,35]. Most of the protocols, however, did not perform repetitive assessments. To implement miRNA as a prognostication tool for long-term outcomes, it is important to appropriately design future protocols and include repetitive clinical and radiological follow-up of study participants. Furthermore, to assess the full spectrum of miRNA involvement in stroke pathology, serial blood testing for miRNA expression levels should be considered. Repetitive NIHSS assessment would be representative of patients’ potential for recovery and neurological improvement. This, however, should be combined with further scanning to assess the final infarct volume. The size of ischemic lesions can evolve over time, depending on various factors such as the extent of edema, reperfusion, progression of ischemia, or secondary hemorrhagic transformation. During the acute period of IS diffusion, the weighted imaging (DWI) sequence demonstrates the intracellular edema that develops within the first hours or even minutes after stroke onset [53]. Irreversible changes to brain tissue can be seen later (3–8 h from symptoms onset) as increased signal intensity on T2-weighted imaging [54]. The reversibility of intracellular edema is highly variable and associated with the level of recanalization [55]. Therefore, the implementation of repetitive clinical and MRI assessment combined with miRNA expression testing in future study protocols could become a tool for appointing potential miRNA markers for reversible and irreversible brain damage in the course of IS. Interestingly, none of the included studies used non-contrast computed tomography (NCCT) for infarct volume assessment. IS lesion volume measurements based on NCCT were proven reproducible and reliable [56,57]. Due to feasibility, common access to NCCT, and economic aspects, we can consider using it in future protocols.

The present SR aimed to assemble available data on the association between miRNA expression and clinical aspects of IS. Several limitations of our work need to be listed. Included studies have not reported a consistent method of miRNA testing. Some of the research used both plasma and serum assessment of miRNA expression; others performed tests on miRNA extracted from circulating blood cells (lymphocytes, neutrophils). The fact that numerous fragments of miRNA were assessed in analyzed studies may be associated with an additional possibility of bias. The clinical deficit was measured primarily using the NIHSS, and the assessment timing was similar (first 24 h after admission or IS onset). However, only a small number of studies provided a follow-up neurological examination. Finally, some of the research lacked data on specific types of IS treatment administered, which may additionally influence the testing results due to potential reperfusion injury in cases where thrombolysis or mechanical thrombectomy were performed [58,59]. We are aware that this SR would additionally benefit from statistical analysis. It is worth mentioning that despite the fact that all of the studies addressed the correlation between miRNA expression and neurological deficit, the specified primary outcomes differed. We have decided that due to the diversity in study methodologies, a narrative synthesis would provide a more comprehensive and contextually appropriate interpretation of our findings without accidentally distorting the results. Considering all of the aspects presented above, the cautious analysis of the included studies provided a sound and comprehensive summary of the available clinical data on the association between the clinical features of IS and miRNA expression in stroke patients.

## 5. Conclusions

miRNA expression in IS patients is significantly correlated to important stroke features, such as clinical severity, infarct volume, systemic inflammatory markers, prognosis, stroke etiology subtype, and the risk of stroke recurrence. After analyzing the available data, we have identified a set of possible miRNA fragment candidates that may be used in stroke diagnostics and have the potential to form a basis for developing future treatment protocols. The research included in the present SR indicates that miRNA expression, particularly miR-125b-5p, miR-143, miR-146b, and miR-218, with positive correlation, and miR-21, miR-93, miR-29b, miR-126, and miR-130, with negative correlation, may be significantly associated with clinical severity, infarct volume and inflammation in IS. More prospective, properly designed protocols are needed to confirm these findings and precisely assess the role of miRNA expression in the course of stroke. Future research in this area must include consistent methods of miRNA expression testing, optimized clinical assessment, and brain imagining schedules.

## Figures and Tables

**Table 1:** Data extraction form.

Lp.	Author, Year	Study Design	Study Group (*n*)	Patient Characteristics	miRNA Studied	Primary Outcome	Secondary Outcome	Results	Follow-Up Period
1.	Chen, 2017 [16]	Case control, prospective	83	50 subjects, 33 controls; AIS patients <72 h from stroke onset	miR-223 Exosomes	NIHSS, infarct volume	TOAST classification, OCSP	- No difference in miR-223 level among TOAST subtypes; - A positive correlation was found between miR-223 and NIHSS; - No correlation between miR-223 and infarct volume.	72 h from onset; no further follow up
2.	Chen, 2018 [17]	Case control, prospective	230	128 subjects, 102 control; AIS patients <24 h from onset	miR-146b	NIHSS, infarct volume	correlation between miR-146b and CRP and IL-6 levels	- Positive correlation between miR-146b and CRP and IL-6; - miR-146b positively correlated with both infarct volume and NIHSS.	24 from onset
3.	Ji, 2016 [18]	Case control, prospective	131	65 subjects, 66 controls; AIS patients <24 h from onset	miR-9 miR-124	Level of miR-9 and miR-124 expression	Correlation of miR and NiHSS, infarct volume, and IL-6	- Both positively correlated to NIHSS, infarct volume, and IL-6.	24 from onset
4.	Jin, 2018 [19]	Case control, prospective	296	148 subjects, 148 controls; AIS patients	miR-126 miR-130a miR-222 miR-218 miR-185	Predicting AIS risk	Correlation of miR with NIHSS, TNF alfa, IL1beta, Il-6, and Il-10	- Levels of miR-222, -218, and -185 were elevated in AIS; - miR-126 and miR-130 were negatively correlated to NIHSS; - miR-218 was positively correlated to NIHSS; - miR-126 and miR-130 were negatively correlated to TNF alfa, IL1beta, and Il-6; - miR-126 and miR-130 correlate with lower disease risk.	No follow up
5.	Jin, 2017 [20]	Case control, prospective	216	106 subjects, 110 controls; AIS within 24 of stroke onset	miR-126 miR-130a miR-378 miR-222 miR-218 miR-185 miR-206 miR-101	miR expression	Correlation with NIHSS	- miRs 126, 130a, 222, 218, and 185 may serve as biomarkers for AIS; - miRs 126, 130a, and 378 declined; - miRs 222, 218, and 185 elevated; - miRs 126, 378, 101, 222, 218, and 206 were correlated with NIHSS.	24 from onset
6.	Liang, 2020 [21]	Case control, prospective	136	76 subjects, 60 controls; AIS patients	miR-448	miR expression	Correlation between miR and NIHSS/ stroke type	- miR-488 was not significantly different among groups of different etiology; - miR-448 expression was elevated in the AIS group; - Significant positive correlation between miR-488 and NIHSS.	No follow up
7.	Liang, 2016 [22]	Case control, prospective	199	102 subjects, 97 controls; AIS patients <72 h from stroke onset	miR-34a-5p	Expression of miR-34a-5p	Correlation between miR expression and NIHSS and infarct volume	- Overexpression of miRNA-34a-5p in acute ischemic stroke patients; - Negative association between miRNA-34a-5p and NIHSS and infarct volume.	72 h
8.	Liu, 2015 [23]	Case control, prospective	42	31 subjects, 11 controls; AIS within 24 of stroke onset	miR-9 miR-124 miR-219	miR expression	Correlation between miR expression and NIHSS, stroke volume and inflammatory parameters	- miR-124 was significantly decreased, miR-9 was decreased in patients with larger stroke, and there were no significant changes in serum miR-219; - miR-124 and miR-9 were negatively correlated with infarct volume and CRP; - All miRs correlated negatively with MMP9.	24 h
9.	Liu, 2021 [24]	Case control, prospective	270	170 subjects, 100 controls; AIS patients	miR-21	expression of lnc-MEG3 and miR-21	Correlation between miR-21 and Il-6, Il-17a, TNFalfa, NIHSS, and accumulating recurrence rate	- miR-21 was reduced in AIS; - miR-21 was negatively correlated with TNF-α, IL-17A, NIHSS, and accumulating recurrence rate in AIS.	No follow up
10.	Liu, 2019 [25]	Case control, prospective	65	40 subjects, 25 controls; AIS patients	miR-128	Expression of miR-128	Correlation between miR and NIHSS at 7 days, mRS at 90 days, and infarct volume	- miR128 expression was higher in AIS; - miR-128 levels in circulating lymphocytes were positively correlated with the infarction volume, NIHSS scores at 7 days, and mRS at 90 days; - miR-128 levels in the circulating neutrophils and plasma were not correlated with the infarction volume and NIHSS.	90 days
11.	Ma, 2022 [26]	Case control, prospective (+animal)	100	60 subjects, 40 controls; AIS 6 h after stroke onset	miR-29b	Expression of miR-29b	Correlation with. NIHSS	- miR-29b in NEU is downregulated in IS; - Negative correlation between NEU miR and NIHSS score at admission.	6 h
12.	Mostafa, 2023 [27]	Case control, prospective	80	40 subjects, 40 controls, AIS treated with rtPA, assessed on admission and daily throughout 7 days and after 3 months	miR-125b-5p	miR expression	Correlation with stroke type, NIHSS, infarct volume and mRS	- No difference in miR expression in different stroke types; - Positive correlation between miR expression and NIHSS and infarct volume at day 7; - Higher miR expression in patients with complications after rtPA and with poor outcome.	3 months
13.	Rahmati, 2020 [28]	Case control, prospective	104	52 subjects, 52 controls; AIS patients	miR-602	Expression of S100B and miR-602	Correlation between miR expression and NIHSS and survival at 3 months	- Lower miR-602 in AIS patients; - miR-602 was elevated in patients with a higher NIHSS score.	3 months
14.	Song, 2020 [29]	Case control, prospective	Unknown number of patients and controls	AIS patients	miR-152-3p	miR expression	Correlation between miR expression and NIHSS, stroke type, and stroke phase	- miR-152-3p in patients with AIS was significantly lower; - A decrease in exosome miR-152-3p level is significantly related to the severity of endothelial injury; - The lowest level of exosome miR-152-3p was found in large- artery atherosclerosis; - miR-152-3p level was significantly lower in the acute phase than in the chronic phase.	No follow up
15.	Wang, 2021 [30]	Case control, prospective	176	88 subjects, 88 controls; AIS patients	miR-9-5p miR-128-3p	miR expression	Correlation of miR and mRS and patient comorbidity	- Expression of the miRs was elevated in AIS patients; - miRNAs were positively correlated with the prognostic MRS scores; - Levels of miR-9-5p and miR-128-3p were correlated with BP, BMI, LDL levels, hypertension, and hyperlipidemia.	No follow up
16.	Wand, 2015 [31]	Case control, prospective (+animal)	117l	58 subjects, 59 controls; AIS patients, sampule taken < 72 h after onset	miR-29b	miR expression in white blood cells	Correlation between miR29b and NIHSS, mRS, and infarct volume	- miR-29b was significantly downregulated in stroke patients; - miR-29b negatively correlated with NIHSS and infarct volume.	3 months
17.	Xiang, 2017 [32]	Case control, prospective	125	46 subjects without rtPA, 40 subjects with rtPA, 39 controls; AIS patients 24 h post onset	let-7i miR-15a	miR expression	Correlation between miR and NIHSS	- miR-15a expression decreased by 0.5-fold in the rt-PA group compared to that in the non-rt-PA group; - No significant correlation between miR15a and NIHSS.	No follow up
18.	Sue, 2018 [33]	Case control, prospective	120	65 subjects, 55 controls; AIS patients within 24 h of AIS	miR-9	miR-9 expression	Correlation between miR09 and NIHSS, inflammatory factors, OGD- induced neuronal injury	- miR-9 was highly expressed in the serum of patients with AIS; - miR-9 was positively correlated with NIHSS; - serum miR-9 in AIS patients had a positive correlation with serum Il-1beta, TNFalfa, and IL-8.	No follow up
19.	Zhong, 2021 [34]	Case control, prospective (+animal)	128	89 subjects, 39 controls; AIS patients	miR-497	miR-497 expression	Correlation of miR with neurological function (NIHSS) and oxidative stress (SOD, MDA); association with prognosis of CIS	- miR-497 negatively correlated with the NIHSS and MDA concentration; - Positively related to SOD concentration; - Higher prognostic mortality in the low miR-497 group.	3 years
20.	Zhou, 2018 [35]	Case control, prospective	100	50 subjects, 50 controls; AIS patients within 24 h of AIS	miR-134	miR-134 expression in AIS	Correlation between miR expression and NIHSS, prognosis, infarct volume and inflammatory factors (IL-6, CRP)	- High expression of miR134 in AIS patients - miR134 was positively correlated with NIHSS, infarct volume and worse prognosis; - miR134 was positively correlated with IL-6 and cRP.	3 months
21.	Zhou, 2021 [36]	Case control, prospective	216	108 subjects, 108 controls; AIS patients at 24 h, 48 h, and 72 h	miR-124	miR-124 expression	Correlation between miR expression and inflammatory factors and prognosis in GOS	- miR-124 expression was poorly expressed in the serum of ACI patients; - miR expression was not correlated to infarct classification, infarct size, low-density lipoprotein level, and homocysteine level; - miR-124 expression was negatively correlated with IL-6, IL-8, and CRP; - Low expression of miR-124 was positively correlated with the poor prognosis.	30 days
22.	Zhu, 2019 [37]	Case control, prospective	340	170 subjects, 170 controls; AIS within 24 h of onset	miR-143	miR-143 and circ-DLGAP expression	Correlation between miR143 and NIHSS and inflammatory factors	- miR-143 was positively associated with NIHSS score, CRP, ESR, TNF-α, IL-1β, IL-6, IL-8, IL-17, and IL-22.	No follow up
23.	Gus, 2020 [38]	Case control, prospective	235	170 elderly AIS patients, 65 control AIS patients; divided into groups based on mRS and NIHSS score	miR-24 miR-29b	miR-24 and miR-29b expression	Correlation between miR expression and NIHSS and prognosis	- miR-24 and miR-29b in the AIS group were significantly lower than those in the healthy control group - miR-24 and miR-29b in the poor neural function prognosis group were significantly lower than those in the good neural function prognosis group - Expression levels of serum miR-24 and miR-29b in the severe group were significantly lower than those in the mild and moderate groups; - Expression levels of serum miR-24 and miR-29b were negatively correlated with NIHSS score.	No follow up
24.	He, 2019 [39]	Prospective cohort study	84	84 subjects; AIS patients that received rtPA	miR-124-3p miR-125b-5p miR-192-5p	miR expression	Correlation between miR expression and outcome and stroke severity	- miR-124-3p, miR-125b-5p, and miR-192-5p levels were higher in patients with unfavorable outcomes than in patients with favourable outcomes - miR-124-3p and miR-125b-5p were closely associated with stroke severity.	3 months
25.	He, 2019 [40]	Prospective cohort study	94	94 subjects; AIS 24 h after thrombolysis with or without endovascular treatment	miR-125b-5p miR-206	Correlation between miR and stroke severity (NIHSS, infarct volume) and outcome	Correlation between miR expression and hemorrhagic transformation	- miR-125b-5p and miR-206 levels were correlated with NIHSS and infarction volumes; - miR-125b-5p levels were significantly higher in patients with an unfavorable outcome; - No association between miRNAs and ICH.	90 days
26.	Ma, 2019 [41]	Prospective cohort study	?	AIS patients within 6 h from onset	miR-93 (plasma and neutrophil)	Expression of plasma and neutrophil miR-93	Correlation between miR-93 and stroke severity and inflammation	- miR-93 levels in plasma and neutrophil detected by real-time PCR were evidently reduced in AIS patients; - miR-93 was not correlated with infarct volume and NIHSS; - miR-93 levels in plasma and neutrophils of AIS patients were negatively correlated with the expression of TNF-α and IL-10; - Neutrophil miR-93 was positively correlated with Barthel index 7 days post stroke.	No follow up
27.	Rainer, 2016 [42]	Prospective cohort study	84	84 AIS patients; AIS patients presenting to the ER within 24 from onset	miR124-3p miR16	3 month mortality	Post-stroke mRS	Plasma miR-124-3p concentrations were elevated in patients who died compared to patients who survived; - mir124 was higher in patients with a 3-month mRS > 2 compared to patients with mRS ≤ 2; - Higher miR-16 concentrations in patients who survived than in patients who died; - miR16 concentrations were lower in patients achieving mRS > 2 than in patients with mRS ≤ 2.	3 months

AIS—acute ischemic stroke; NIHSS—National Institutes of Health Stroke Scale; TOAST—Trial of Org 10,172 in Acute Stroke Treatment; OCSP—Oxfordshire Community Stroke Project; mRS—modified Rankin Scale, GOS—Glasgow Outcome Scale, rtPA—Recombinant Tissue Plasminogen Activator.

**Table 2:** Outcome summary table.

Outcome	Relationship Between miRNA Expression and Selected Outcomes		
	Positive	Negative	No Association
**Clinical severity**	miR-9* miR-101 miR-124* miR-125b-5p miR-128 miR-134 **miR-143** miR-146b miR-185 miR-206 **miR-218** miR-222 miR-223 miR-488 miR-602	miR-9* miR-16 **miR-21** miR-24 **miR-29b** miR-34a-5p miR-124* miR-126 **miR-130** miR-152-3p miR-378 miR-497	miR-9* miR-15a miR-93 miR124* miR-128* miR-219
**Infarct volume**	miR-9* miR-124* **miR-125b-5p** miR-128* miR-134 miR-146b miR-206	miR-9* miR-29b miR-34a-5p miR-124*	miR-93 miR-124* miR-128* miR-223
**Systemic inflammatory markers**	miR-9* miR124* miR-134 miR-143 miR-146b miR-497	miR-9* miR-21 miR-93 miR-124* miR-126 miR-130	miR-9* miR-124*
**Prognosis**	miR-9 miR-24 miR-124	miR-134	
**Etiology subtype**		miR152-3p	miR-124 miR-223 miR-488
**Risk of stroke recurrence**		miR-21 miR-126 miR-130	

## Data Availability

The original contributions presented in the study are included in the article, further inquiries can be directed to the corresponding author.
